# Workplace sexual harassment and depressive symptoms: a cross-sectional multilevel analysis comparing harassment from clients or customers to harassment from other employees amongst 7603 Danish employees from 1041 organizations

**DOI:** 10.1186/s12889-017-4669-x

**Published:** 2017-09-25

**Authors:** Maria K. Friborg, Jørgen V. Hansen, Per T. Aldrich, Anna P. Folker, Susie Kjær, Maj Britt D. Nielsen, Reiner Rugulies, Ida E. H. Madsen

**Affiliations:** 1 National Research Centre for the Working Environment, Lerso Parkalle 105, DK-2100 Copenhagen, Denmark; 2 COWI A/S, parallelvej 2, DK-2800 Kongens Lyngby, Denmark; 3 National Institute of Public Health, University of Southern Denmark, Øster Farimagsgade 5A, DK-1353 Copenhagen K, Denmark; 4 Department of Public Health, University of Copenhagen, Copenhagen, Denmark; 5 Department of Psychology, University of Copenhagen, Copenhagen, Denmark

**Keywords:** Sexual harassment, Depressive symptoms, Occupational health, Multilevel modeling, Effect modification

## Abstract

**Background:**

Previous research has reported that sexual harassment can lead to reduced mental health. Few studies have focused on sexual harassment conducted by clients or customers, which might occur in person-related occupations such as eldercare work, social work or customer service work. This study examined the cross-sectional association between sexual harassment by clients or customers and depressive symptoms. We also examined if this association was different compared to sexual harassment conducted by a colleague, supervisor or subordinate. Further, we investigated if psychosocial workplace initiatives modified the association between sexual harassment by clients or customers and level of depressive symptoms.

**Methods:**

We used data from the Work Environment and Health in Denmark cohort study (WEHD) and the Work Environment Activities in Danish Workplaces Study (WEADW) collected in 2012. WEHD is based on a random sample of employed individuals aged 18–64. In WEADW, organizational supervisors or employee representatives provided information on workplace characteristics. By combining WEHD and WEADW we included self-reported information on working conditions and health from 7603 employees and supervisors in 1041 organizations within 5 occupations. Data were analyzed using multilevel regression and analyses adjusted for gender, age, occupation and socioeconomic position.

**Results:**

Exposure to workplace sexual harassment from clients or customers was statistically significantly associated with a higher level of depressive symptoms (2.05; 95% CI: 0.98–3.12) compared to no exposure. Employees harassed by colleagues, supervisors or subordinates had a higher mean level of depressive symptoms (2.45; 95% CI: 0.57–4.34) than employees harassed by clients or customers. We observed no statistically significant interactions between harassment from clients and customers and any of the examined psychosocial workplace initiatives (all *p* > 0.05).

**Conclusions:**

The association between sexual harassment and depressive symptoms differed for employees harassed by clients or customers and those harassed by colleagues, supervisors or subordinates. The results underline the importance of investigating sexual harassment from clients or customers and sexual harassment by colleagues, supervisors or subordinates as distinct types of harassment. We found no modification of the association between sexual harassment by clients or customers and depressive symptoms by any of the examined psychosocial workplace initiatives.

**Electronic supplementary material:**

The online version of this article (doi:10.1186/s12889-017-4669-x) contains supplementary material, which is available to authorized users.

## Background

According to the EU Directive of 2006, [1] sexual harassment is any form of unwanted verbal, non-verbal or physical conduct of a sexual nature which occurs with the purpose or effect of violating the dignity of a person, in particular when creating an intimidating, hostile, degrading, humiliating or offensive environment. Sexual harassment is a gendered phenomenon as women are more likely to be exposed compared to men. A survey among Danish employees shows that 5.1% of women aged 18–64 years have been exposed to sexual harassment compared to 1.2% of men [2]. The same survey also showed that the prevalence was higher among employees working in health care: the prevalence of sexual harassment across all jobs was 3.1%, and for health care workers it was 16.4% [2]. Another study of 8064 Danish employees showed that in the health care sector, sexual offensive behaviors at work were 3.5 times more often reported than the national average of all other jobs [3, 4]. Among health care workers, sexual harassment is most often conducted by clients or customers [3, 5, 6].

Research reports that workplace sexual harassment has negative consequences such as decreased job satisfaction, long-term sickness absence, and mental health problems such as depression and anxiety [7–11]. One of the most commonly studied consequences of sexual harassment is its effects on mental health [7, 11]. Most research has focused on sexual harassment from colleagues, supervisors or subordinates while sexual harassment conducted by clients or customers has received limited interest, despite studies showing such harassment is frequent [12–16]. Small studies of occupation-specific samples find associations between sexual harassment by clients or customers and adverse mental health conditions including depression and burnout [13, 17–21] but there is a paucity of larger cross-occupational studies in the field. A reason for the limited interest in sexual harassment from clients or customers may be that organizations may normalize and neglect the seriousness of this act [3, 7, 22]. At some workplaces there is a tendency to consider sexual harassment from clients or customers as a part of the job and not as a potentially harmful experience [3, 22]

### Sexual harassment by clients or customers

Sexual harassment conducted by clients or customers may occur in person-related occupations, i.e. jobs that require interactions with clients or customers [23, 24]. Examples of person-related work include care work - caring for individuals who are elderly, ill, or disabled - social work, and customer service work. Harassment conducted by clients or customers may differ from sexual harassment conducted by colleagues, supervisors or subordinates. Employees in person-related professions may have to navigate between their own perception of unacceptable behavior and an organizational expectation that it is part of professional competency to manage difficult clients or customers [3, 22, 25–27]. Often it is not well defined in the organization what kinds of behavior are to be accepted [3, 22, 28–30]. Literature indicates that organizations may refrain from explicitly taking on the responsibility for making guidelines and policies regarding sexual harassment from clients and customers [3, 22, 28–30]. Consequently, the responsibility of setting boundaries and evaluating the behavior of a client or customer to determine if the behavior is acceptable or not is left to individual employees [25]. In recent years there has been a gradual change in the conception of professional practice in person-related jobs [3, 23, 24]. With respect to employees working with institutionalized clients, there is an increasing emphasis of the client’s self-determination [3, 27]. The sexual needs of clients should not be ignored or rejected but accepted and thus a practice is constituted where the professional may potentially harm the client by ignoring of sexual utterances [3, 22, 27]. Also sexual harassment in person-related professions may often be explained by client dementia or cognitive impairment, [25, 27, 31] making the organization conclude that the harassment is unintended and thus unharmful. In this way sexual harassment by clients might be normalized as an inevitable working condition and not as sexual harassment [22]. This normalization may not occur if the perpetrator is a colleague, supervisor or a subordinate.

In person-related professions, it may be difficult to distinguish between inappropriate sexual behavior from clients and work-related responsibilities. With intimate care sometimes being part of work-related responsibilities, employees are more likely to be confronted with aspects related to sexuality and sexual needs of the patients, e.g. if the patient gets an erection during bathing. It may, in many situations, be challenging to distinguish if a client’s behavior is acceptable or not, for instance when patients are cognitively impaired and not able to understand the consequences of their actions. In eldercare, employees are also often alone with the client in his or her home making it difficult for the employee to set boundaries. Over time, the employee may perceive the responsibility of setting boundaries as something that strains his or her coping resources [13, 32]. Following stress theories this might result in negative mental health outcomes such as depressive symptoms and depression [32–34]. Among employees working with customers similar tendencies as described above may apply [19, 35]. Employees working with customers have to navigate in an organizational climate, where customer’s satisfaction is a main priority [16, 19, 36, 37]. Thus, as with clients, sexual harassment might not be an organizational concern but a matter of employees setting boundaries and at the same time react on customers’ needs [3, 16, 24].

Employees in person-related professions not only have to set boundaries. They also have to relate to the potential risk of recurrent incidences of sexual harassment. Sexual harassment is more often recurrent when conducted by clients or customers compared to sexual harassment by colleagues, supervisors or subordinates [15, 38–40]. Recurrent exposure can be explained by the specific nature of person-related work [38]. First, the same client or customer is likely to harass an employee more than once, if there is a continued contact between the employee and the client or customer, as is often the case at hospitals, psychiatric institutions, and in the elder care. Second, employees may experience sexual harassment from more than one client or customer during their career, if they stay in the same job were the risk of exposure is higher than for the general working population [13, 15].

The aims of this study are: First, to examine the cross-sectional association between exposure to sexual harassments by clients or customers and the level of depressive symptoms; Second, to examine if the association between sexual harassment and depressive symptoms is different when the perpetrator is a client or customer compared to a colleague, supervisor or subordinate; Third, to examine if different psychosocial workplace initiatives (activities to prevent sickness-absence, access to treatment, evaluation of the psychosocial work environment) may modify the association of sexual harassment by clients or customers with level of depressive symptoms in a large cross-occupational sample of 7603 employees from Denmark.

## Methods

### Study design and population

Data were derived from two sources: 1) The Work Environment and Health in Denmark cohort study (WEHD) and 2) the Work Environment Activities in Danish Workplaces Study (WEADW). WEHD is based on a random sample of employed individuals aged 18–64. The study is designed to document developments in work environment and health in the Danish working population, and contains comprehensive self-reported work environment data. Data are collected every second year from 2012 to 2020, using either postal or web-based questionnaires. The present analyses were based on data from 2012, where the overall response rate was 50.8%, and there were 7603 respondents from WEHD who were employed within 1041 organizations participating in WEADW. In the WEADW survey, organizational supervisors or employee representatives supplied information on the organizational work environment of the participating workplaces. The workplaces were selected to represent a range of occupational sectors, including building and construction, private service, knowledge work, care work and industrial work.

**Fig. 1:**
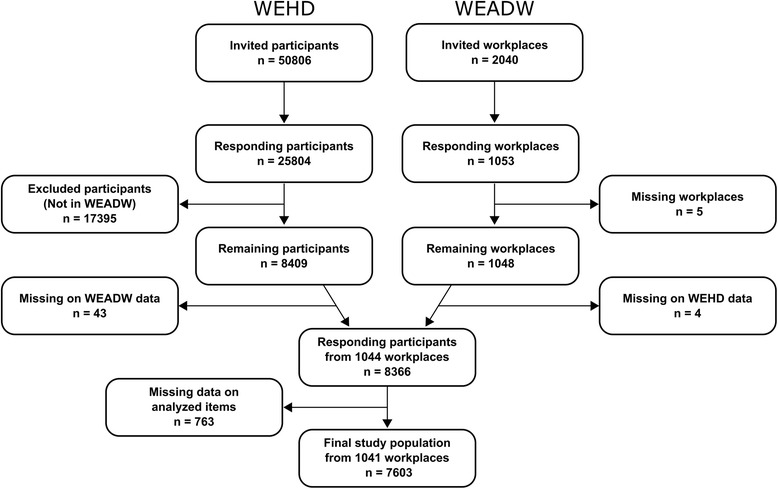
Flow chart of the exclusion processes for the study population included in the final statistical analyses

For WEADW, 2040 organizations were invited to participate and 1053 organizations responded (51.6%). A total of 8409 (53%) employees within these organizations replied to WEHD. Each participating organization could have up to four answers on WEADW as this questionnaire were sent to both organizational supervisors, employee representatives, work environment representatives and owners of the organization. For the present analyses only one answer per organization were included with the following prioritization: 1) owners, 2) organizational supervisors, 3) employee representatives, 4) work environment representatives, and 5) other. We chose this hierarchical order, as we assume owners and supervisors to have the most comprehensive knowledge of the respective organization. Five of the responding organizations had missing data on the psychosocial workplace initiatives and were thus excluded. By merging the two datasets, we identified 8366 employees from 1044 organizations. We excluded employees with missing data on any of the analyzed items (sexual harassment, the MDI, socioeconomic status and occupational group) (*n* = 763). The final study population consisted of 7603 participants within 1041 organizations. Figure 1 summarizes the selection of the study population.

### Measurements

#### Workplace sexual harassment

Workplace sexual harassment was measured with the following item: “Have you been exposed to sexual harassment at your workplace during the last 12 months?” The response categories were “yes, daily”, “yes, weekly”, “yes, monthly”, “yes, rarely” or “never”. The item measured perceived exposure to sexual harassment and no definition of sexual harassment was given in the questionnaire. Participants responding “yes” were asked to report if the perpetrator was a “colleague”, “supervisor”, “subordinate” or “a subject not employed at the workplace of the respondent, e.g. clients or customers”. Given the distribution of participants, with relatively few respondents being frequently exposed, we categorized exposure to workplace sexual harassment as “yes, from clients/customers” (exposed to sexual harassment and reporting client/customer as perpetrator), “yes, from others” (exposed to sexual harassment and reporting colleague, supervisor or subordinate as perpetrator), or “no” (not exposed to sexual harassment).

#### Depressive symptoms

Depressive symptoms were assessed with the Major Depression Inventory (MDI) [41, 42]. The MDI includes 10 items (12 questions) that cover the ICD-10 (International Classification of Diseases 10th revision) and DSM-IV (Diagnostic and Statistical Manual of Mental Disorders fifth edition) symptoms of depression and major depression. The items are measured in frequency, using the past 2 weeks as the time frame [41, 42]. The questions and response categories are shown in Table 1. Responses were scored 0–5 and summed, yielding a scale from 0 to 50, with higher scores indicating a higher level of depressive symptoms. For a sensitivity analysis we dichotomized the MDI scale score into probable depression or not by the cut-off ≥20 in accordance with previous studies indicating this as the most accurate cut-off score for probable depression [43, 44]. Bech and colleagues (2001) have validated the MDI as a measure of depression using SCAN (Schedules for Clinical Assessment in Neuropsychiatry) interviews [41].

**Table 1:** Items of the Major Depression Inventory

Major Depression Inventory	
How much of the time	
1. Have you felt low in spirits or sad?	
2. Have you lost interest in your daily activities?	
3. Have you felt lacking in energy and strength?	
4. Have you felt less self-confident?	
5. Have you had a bad conscience or feelings of guilt?	
6. Have you felt that life wasn’t worth living?	
7. Have you had difficulty in concentrating, e.g. when reading the newspaper or watching television?	
8. Have you felt very restless?	
9. Have you felt subdued or slowed down?	
10. Have you had trouble sleeping at night?	
11. Have you suffered from reduced appetite?	
12. Have you suffered from increased appetite?	

Response categories: “all the time”, “most of the time”, “slightly more than half the time”, “slightly less than half the time”, “some of the time”, “at no time”

#### Psychosocial workplace initiatives

Psychosocial workplace initiatives were measured with three items: 1) “within the last year, has the organization implemented activities to prevent sickness-absence?” We dichotomized the response options into “yes” (“to a high degree”/“partly”) and “no” (“to a low degree”/“no”/“do not know”). Organizations answering “not relevant” were excluded from this specific analysis. 2) “Within the last year, were employees offered health insurance to cover treatment by specialists (physiotherapist, psychologist or similar)?” This item had four response categories, which we combined into “yes” (“yes, offered during working hours”/“yes, offered outside of working hours”) and “no” (“no”/“do not know”). 3) “Within the last three years, has the organization evaluated the psychosocial work environment?” The question had three response categories, which we dichotomized into “yes” (“yes”) and “no” (“no”/“do not know”). We chose the above-mentioned psychosocial workplace initiatives as we expected them to potentially be able to buffer any negative mental health consequences of sexual harassment. Access to treatment by a psychologist might for instance help employees cope with harassment. Thus the association between harassment and depressive symptoms might be weaker in workplaces that offer access to such treatment. This would be likely given data supporting effects of psychotherapy on depression [45]. Also, organizations which evaluate their psychosocial working conditions might be more likely to uncover problems relating to sexual harassment and initiate interventions to prevent and manage this exposure. Furthermore, organizations implementing activities to prevent sickness absence might have implemented initiatives dealing with sexual harassment, if harassment was identified as a problem within the organization. If such initiatives to manage sexual harassment were successful, the effects of sexual harassment on employee mental health might be weakened in organizations with such interventions compared to organizations without such interventions.

### Statistical analyses

We analyzed data using Multilevel Modeling given the hierarchical structure of our data. In our dataset employees (level 1) are nested within organizations (level 2), resulting in two hierarchical levels. By using a mixed model with random intercepts across organizations, we accounted for correlated observations from participants being employed in the same organization [46–48]. Allowing the parameter determining the association between sexual harassment and depressive symptoms to vary between organizations (random slope model) made only negligible changes in the results in all but one case (mentioned in results). Therefore we report results from a fixed slope model.

We first examined how depressive symptoms were associated with sexual harassment from clients or customers in an analysis comparing participants with this exposure to participants not exposed to sexual harassment. We excluded those harassed by colleagues, supervisors or subordinates. Subsequently, we examined the mean difference in depressive symptom level between participants exposed to harassment from colleagues, supervisors or subordinates compared to participants harassed by clients or customers. Finally, again comparing participants harassed by clients or customers to non-exposed participants and excluding participants harassed by colleagues, supervisors or subordinates, we tested whether the psychosocial workplace initiatives modified the association between sexual harassment from clients or customers and depressive symptoms. We tested for statistical interaction (departure from additivity) between each workplace initiative and exposure (sexual harassment from clients or customers) in their association with depressive symptoms by including an interaction term between the exposure and the respective workplace initiative. This analysis was conducted separately for each psychosocial workplace initiative. We adjusted all associations for potential confounding by sex, age, socioeconomic position and occupation, as these factors are related to depressive symptoms [49–52] and workplace sexual harassment [3, 8, 38, 53]. All analyses used a level of statistical significance of *P* < 0.05 and *P*-values were calculated using Restricted Maximum Likelihood estimation. Data were analyzed using SAS, version 9.4 (SAS Institute, Cary, NC).

#### Potential confounders

We derived sex and age from the participants’ civil registration number [54], while information on occupation were derived from the job register. We classified respondent’s occupation into five main groups: “Knowledge work” (e.g. working in public administration or education), “Private service” (e.g. working in supermarkets or restaurants), “Care work” (e.g. working in hospitals or residential care), “Industrial work” (e.g. working in manufacturing), or “Building and construction” (e.g. working in bricklaying or civil engineering). The coding of subgroups is presented in Additional file 1: Appendix 1, and is based on the Danish version of EU’s nomenclature (NACE, Statistical classification of economic activities in the European Community), which is a statistical classification of economic activities [55]. Socioeconomic status was determined from group of occupation following the Danish version of the International Standard Classification of Occupations approved in 2008 (ISCO-08) [56]. This classification organizes jobs on the basis of the skills needed to perform them [56].

#### Sensitivity analyses

We conducted three sets of sensitivity analyses: First, we tested the examined associations for care workers only. This analysis was conducted because this article was written as part of a project focusing especially on sexual harassment conducted by clients, amongst care workers. Second, we tested whether the examined associations were different if the outcome was clinical depression instead of depressive symptoms. Third, we conducted a gender stratified analyses, because some previous analyses suggest that sexual harassment may have gender specific effects [57]. Gender stratification was not part of the main analyses of the article, because of the low number of men exposed to sexual harassment in the sample, yielding a high level of statistical uncertainty in this part of the analyses.

## Results

### Descriptive analyses

Table 2 presents the characteristics of the participants stratified by sexual harassment from clients/ customers and sexual harassment from colleagues/supervisors/subordinates. The participants had a mean age of 46 years and the largest occupational group was care work (28.8%), whereas 24.9% were employed in knowledge work, 17.9% in industrial work, 16.3% in private service, and 12.0% in building and construction. Slightly more women (54.1%) than men (45.9%) participated. Of the participants, 2.4% were exposed to sexual harassment by clients/customers. Women (4.1%) were more likely to be exposed than men (0.3%). In comparison, 1.0% was exposed from colleagues/ supervisors/subordinates, and this exposure was reported by 1.2% of the women, but only 0.9% of male respondents. Participants employed in care work were more often exposed to sexual harassment from clients/customers (6.9%) than participants employed in private service (1.0%), knowledge work (0.7%), building and construction (0.2%), and industrial work (0.0%). Older employees were generally less like than younger employees to be exposed from clients/customers. Among the participants in private service, 1.8% was exposed to harassment from colleagues/supervisors/subordinates, whereas this percentage was 1.1% in industrial work, 1.1% in building and construction, 0.8% in knowledge work and only 0.7% in care work.

**Table 2:** Baseline characteristics for the study population

	Total		Sexual harassment from clients/customers		Sexual harassment from colleagues/supervisors/subordinates		Not exposed	
	*N (%)*		*N (%)*		*N (%)*		*N (%)*	
Total	7603	(100)	180	(2.4)	79	(1.0)	7344	(96.6)
Sex								
Men	3487	(45.9)	11	(0.3)	31	(0.9)	3445	(98.8)
Women	4116	(54.1)	169	(4.1)	48	(1.2)	3899	(94.7)
Age(Mean = 46.0,SD = 10.9)								
18–29	689	(9.1)	28	(4.1)	22	(3.2)	639	(92.7)
30–39	1352	(17.8)	29	(2.1)	19	(1.4)	1304	(96.4)
40–49	2309	(30.4)	56	(2.4)	20	(0.9)	2233	(96.7)
50+	3253	(42.8)	67	(2.1)	18	(0.6)	3168	(97.4)
Occupation								
Care work	2191	(28.8)	152	(6.9)	16	(0.7)	2023	(92.3)
Knowledge work	1895	(24.9)	13	(0.7)	16	(0.8)	1866	(98.5)
Industrial work	1364	(17.9)	≤ 3		15	(1.1)	1349	(98.9)
Private service	1242	(16.3)	13	(1.0)	22	(1.8)	1207	(97.2)
Building and construction	911	(12.0)	≤ 3		10	(1.1)	899	(98.7)
Socioeconomic status								
Senior manager in concerns, organizations and the public sector	342	(4.5)	4	(1.2)	≤ 3		336	(98.2)
Employee in an occupation acquiring skills on the highest level	2393	(31.5)	42	(1.8)	18	(0.8)	2333	(97.5)
Employee in an occupation acquiring skills on an intermediate level	885	(11.6)	≤ 3		12	(1.4)	876	(98.6)
Employee in an occupation acquiring skills on ground level	3130	(41.2)	129	(4.1)	34	(1.1)	2967	(94.8)
Other employees	853	(11.2)	5	(0.6)	13	(1.5)	835	(97.9)
Has the organization implemented activities to prevent sickness-absence?								
Yes (to a high degree/partly)	6246	(82.2)	159	(2.5)	65	(1.0)	6022	(96.4)
No (to a low degree/ no/do not know)	1228	(16.2)	19	(1.5)	14	(1.1)	1195	(97.3)
Not relevant	129	(1.7)	≤ 3		≤ 3		127	(98.4)
Were employees offered health insurance to cover treatment by specialists (physiotherapy, psychologist or similar?								
Yes (yes, offered during working hours/yes, offered outside of working hours)	5527	(72.7)	129	(2.3)	55	(1.0)	5343	(96.7)
No (no/do not know)	2076	(27.3)	51	(2.5)	24	(1.4)	2001	(96.4)
Has the organization evaluated the psychosocial work environment?								
Yes (yes)	5857	(77.0)	157	(2.7)	65	(1.1)	5635	(96.2)
No (no/do not know)	1746	(23.0)	23	(1.3)	14	(0.8)	1709	(97.9)

≤ 3. Due to restrictions regarding potential person-identifiability of data, counts of less than 3 participants cannot be reported

### Association of sexual harassment and depressive symptoms

Table 3 shows estimated differences and the *P*-value of the multilevel regression analyses. Both types of exposure to sexual harassment were associated with an increased level of depressive symptoms. Compared to employees not exposed to sexual harassment, the mean level of depressive symptoms was 2.05 (95% CI: 0.98–3.12) (*p* = 0.0002) points higher for employees harassed by clients/customers. Employees harassed by a supervisor/colleague/subordinate had a further elevated mean level of 2.45 (95% CI: 0.57–4.34) (*p* = 0.011) points. Applying a random slopes model instead, yielded slightly different results, estimating this mean difference to be 2.70 (95% CI: 0.45–4.95).

**Table 3:** Mean level of depressive symptoms in relation to sexual harassment

Sexual harassment		
	Estimated mean difference	*P* value
Association of sexual harassment and depressive symptoms		
Sexual harassment from clients/customers compared to non-exposed employees (*N* = 7524)	2.05 (95% CI: 0.98–3.12)	.0002
Sexual harassment from colleagues/supervisors compared to Sexual harassment from clients/customers (*N* = 7603)	2.45 (95% CI: 0.57–4.34)	0.011
Effect-modification analyses		
Workplace characteristics	Estimated modification of association	
Has the organization implemented preventive activities to avoid sickness-absence? (*N* = 7395)	0.31 (95% CI: −3.09;3.71)	0.86
Access to treatment (physiotherapy, psychologist or similar)? (*N* = 7524)	−0.52 (95% CI: −2.85;1.81)	0.66
Has the organization analyzed the psychological work environment? (*N* = 7524)	−0.83 (95% CI: −3.95;2.29)	0.60

All estimates are adjusted for gender, age, socioeconomic position and occupation

### Effect modification by psychosocial workplace initiatives

Regarding the psychosocial workplace initiatives as potential modifiers of associations, we found no statistically significant interactions of harassment from clients/customers with any of the examined psychosocial workplace initiatives (all *p* > 0.05). There were also no statistically significant main effects of the examined psychosocial workplace initiatives on the level of depressive symptoms amongst employees (all *p* > 0.05, data available on request).

### Sensitivity analyses

In sensitivity analysis 1, we restricted our sample to care workers and found a mean level of depressive symptoms that was 2.05 (95% CI: 0.83–3.27) (*p* = 0.001) points higher for employees exposed to sexual harassment from clients or customers compared to non-exposed employees. In this analysis, however, there was no statistically significant difference in symptom level between participants harassed by supervisors/colleagues/subordinates compared to participants harassed by clients/customers (mean difference − 1.03(−3.14; 1.08)).

By changing the outcome to clinical depression (sensitivity analysis 2) we found no statistically increased risk (OR = 1.19 (95% CI: 0.72–1.97) (*p* = 0.50) of clinical depression among those harassed by clients/customers compared to non-exposed. For participants harassed by supervisors/colleagues/subordinates we observed a statistically significantly increased risk with an odds ratio of 3.30(1.62–6.73), *p* = 0.001.

When conducting the analyses separately for men and women, results were largely similar to those from the main analysis. The mean difference in depressive symptoms associated with harassment from clients/customers compared to non-exposed participants was 1.36 points (−2.56–5.28) for men and 2.17 (1.00–3.34) for women. The mean difference in depressive symptoms associated with harassment from supervisors/colleagues/subordinates compared to participants harassed by clients/customers was 4.64 points (0.06–9.21) for men and 1.35 (−1.05–3.75) for women.

We found no statistically significant interactions with the examined psychosocial workplace initiatives in any of the sensitivity analyses.

## Discussion

We found that exposure to workplace sexual harassment was associated with a higher level of depressive symptoms compared to employees not exposed to sexual harassment. We also saw a tendency towards a stronger association between sexual harassment and depressive symptoms, when the harassment was conducted by supervisors, colleagues or subordinates compared to clients or customers. This difference, however, was not robust when limiting the sample to participants working in carework. We found no modification of the association between sexual harassment from clients or customers and depressive symptoms by any of the examined psychosocial workplace initiatives.

### Sexual harassment from clients or customers and depressive symptoms

Our finding of an association between workplace sexual harassment from clients or customers and depressive symptoms is in line with occupation specific studies indicating a relationship between sexual harassment from clients or customers and various negative outcomes such as decreased work ability, headache, self-reported stress, and alcohol use [13, 18, 19, 58–60]. However, two recent Danish studies [40, 57] found no statistically significant association with long-term sickness absence. It is possible that sexual harassment from clients may be more related to “softer” indicators of well-being and less severe mental health problems than to more severe indicators such as long-term sickness absence or clinical mental disorders. For employees to stay in person-related professions, they may feel compelled to cope with sexual harassment. Therefore, employees who are able to remain in person-related professions may be less sensitive to exposure to sexual harassment from clients or customers, compared to other employees (healthy worker selection).

Our findings nonetheless underline the importance of identifying methods to prevent both the occurrence of sexual harassment from clients or customers and the development of depressive symptoms following sexual harassment in person-related professions. This is the case as sexual harassment from clients or customers was associated with a higher mean level of depressive symptoms compared to no exposure, although this difference was smaller than that for exposure from colleagues, supervisors or subordinates. Not only are depressive symptoms associated with reduced general well-being limiting social interactions and work ability, employees experiencing depressive symptoms can also be at substantial risk of developing clinically significant mental health conditions such as depression and anxiety [61–63].

### Sexual harassment from colleagues, supervisors or subordinates and depressive symptoms

We found a tendency that employees harassed by colleagues, supervisors or subordinates had a higher mean level of depressive symptoms compared to employees harassed by clients or customers. This result suggests that it may be important to investigate sexual harassment from clients or customers and sexual harassment by colleagues, supervisors or subordinates as two distinct types of harassment. Several factors may be hypothesized to explain the difference in depressive symptoms associated with these two different forms of harassment.

It may be more difficult for employees to react on and report sexual harassment when conducted by a colleague, supervisor or subordinate. Employees harassed by a colleague, supervisor or subordinate may fear job loss, retribution and that the harasser will not receive any penalty [10]. This notion is in line with research showing that only between 5 and 30% of targets to sexual harassment in general file formal complaints, and that employees harassed by a supervisor are less likely to report the conduct [10, 64]. Contrary, in person-related professions it is often recognized that sexual harassment can occur and it may be perceived as a part of the job to manage such situations [3, 5, 16, 65]. It is also possible that harassment from colleagues, supervisors or subordinates may have had a longer duration compared to harassment from clients. We could not include information on duration in the present study, but it is certainly plausible that longer exposure to harassment has more detrimental mental health effects compared to more brief exposure, all else being equal. In addition, when sexual harassment is conducted by a supervisor, a power-perspective must be acknowledged. It is possible that the effects of sexual harassment are more severe in the context of power imbalance. Sexual harassment by supervisors can also reflect sexual coercion, where the supervisor offers bonuses, pay increases, and promotion in return for sexual attention [10, 66, 67]. Further, sexual harassment by a colleague, supervisor or subordinate can be indicative of a poor work environment to a higher extent than sexual harassment by clients or customers. Research suggest that a poor organizational climate (e.g. a climate that tolerates sexual harassment) is an antecedent to sexual harassment and a direct contributor to mental health problems beyond the experience of sexual harassment [10, 67–71]. Also, studies report that perceptions of organizational procedures for dealing with sexual harassment are related to mental health problems [6, 69]. Finally, as stated above, sexual harassment by clients or customers is often normalized as an inevitable working condition [3, 5, 16, 65]. For employees to stay in person-related professions they may feel they have to accept sexual harassment as a part of professional practice. Therefore, a healthy worker selection may be involved, and the employees who are able to remain in person-related professions may be less sensitive to exposure to sexual harassment from clients or customers, compared to other employees. It should be noted, that given the high correlation between job type and exposure from clients or customers versus colleagues, supervisors or subordinates, the results may reflect underlying differences in consequences of sexual harassment within job types, rather than difference associated with the source of harassment. This could not be separated in detail in the present study. Further research looking into the possible differential effects of sexual harassment depending on the source of exposure, seems warranted.

### Psychosocial workplace initiatives

We found no modification of the association between sexual harassment from clients or customers and depressive symptoms by any of the examined psychosocial workplace initiatives. Temporal factors might influence these results. The time frame for the investigated psychosocial workplace initiatives is up to three years as the participating organizations were asked about the implementation of workplace initiatives within the last three years. Consequently, for some participants there can be a considerable time lapse between the implementation of a workplace initiative and the development of depressive symptoms. It is possible that only recent initiatives influence the relation between sexual harassment by clients or customers and depressive symptoms. Also, the investigated psychosocial workplace initiatives were reported by owners, organizational supervisors, employee representatives or work environment representatives making it a possibility that our result are affected by information bias (supervisors over-reporting the implementation of psychosocial workplace initiatives). Further, the potentially modifying psychosocial workplace initiatives examined in this study were relatively broad and non-specific, as we were limited to data collected in the WEADW. Thus our results do not preclude that more specific workplace initiatives targeted sexual harassment may be effective in preventing the occurrence and consequences of sexual harassment by clients or customers (an issue that we are investigating qualitatively as a separate element of a research project of which the present study is part [72]). Workplace initiatives targeted sexual harassment could for instance establish common guidelines and policies and thus provide employees with criteria for acceptable and non-acceptable behavior. In line with this notion, organizational procedures and climate is found to be the strongest predictor of sexual harassment [11]. Another initiative that can possibly prevent negative consequences following sexual harassment by clients or customers is to make social support from colleagues and supervisors, in cases of sexual harassment, a common and accepted practice [7, 11, 67]. Additionally, as sexual harassment from clients and customers can be a working condition [3, 22], educating employees in avoiding and handling risk situations might be relevant. One study indicates that confidence in responding to sexual harassment can be considered as a resource that prevents development of negative mental health consequences [13].

### Strengths and limitations

This is the first study to demonstrate an association between sexual harassment by clients or customers and depressive symptoms in a large cross-occupational sample of employees. Additionally, this study is the first to compare the mental health consequences of sexual harassment depending on the source of exposure. Given the demographic characteristics of this sample (7603 participants from 1041 organizations) the generalizability across the Danish workforce is high. Further, the strengths of this study is the measurement of several important psychosocial workplace initiatives using WEADW, the application of a well-validated rating scale to measure depressive symptoms, and the usage of multilevel data including answers from both employees and organizational supervisors (representatives for the involved organizations). By applying multilevel modelling, the dependence of employees within an organization is considered [46–48].

Some limitations of the study must be noted. First, the cross-sectional design means that temporality cannot be established and consequently we cannot preclude reverse causality. There is a possibility that depressive symptoms can increase the risk of experiencing sexual harassment at work – potentially because subjects with depressive symptoms tend to perceive the environment more negatively than individuals without depressive symptoms [73, 74]. Consequently, the possibility of reverse causality has to be taken into account when interpreting the results. Second, we used self-reported data, and such data are vulnerable to subjective factors and outcome expectations [75]. Also we relied on participant’s ability to recall episodes of sexual harassment during a 12-month period. Asking participants about sexual harassment experienced within a shorter period may have minimized recall bias [76] but might have underestimated the prevalence of sexual harassment. Third, the exposure to sexual harassment was measured using a single item measurement and there is a possibility that participants may under- or over-report the occurrence of sexual harassment. Studies show that healthcare workers tend to under-report violent incidents at work because they interpret such incidents as a part of their job [5, 13, 77].Single item measurements, on the other hand, have been argued to result in over-reporting compared to using specific questionnaires asking about a range of sexually harassing behaviours [78]. In any case, it is important to keep in mind, that the exposure in the present study was behaviors that were perceived and labelled by the respondents as sexual harassment, irrespective of the specific nature of this behavior. Fourth, although we adjusted for several confounders, we lacked information on other confounders that may be associated with depressive symptoms such as history of psychological illness or traumatic life events [59–61, 76]. Fifth, there was substantial non-response amongst both invited participants and workplaces. There is a possibility that this non-response might be selective, This may reduce the generalizability of our findings, in particular to groups less likely to respond, such as young men with shorter education [79, 80], or workplaces with a poorer psychosocial work climate. Sixth, although we analyzed data from a large cross-occupational sample of Danish employees, the number of exposed individuals was relatively low, increasing the statistical uncertainty of the reported estimates. In particular, the sample included a low number of men exposed to sexual harassment. Whilst this likely reflects a lower prevalence of sexual harassment amongst men than amongst women, it should be noted that the reported main associations are largely driven by the association in women. Though we found no strong indications of different associations in men when separating men and women in a sensitivity analysis, there was a large statistical uncertainty of the estimate for men due to the low number of exposed men included.

## Conclusions

This study found that workplace sexual harassment from clients or customers were associated with employee depressive symptoms. The results underline the importance of identifying methods to prevent sexual harassment from clients or customers and to prevent the development of depressive symptoms following this type of harassment. We found no modification of the association by any of the examined psychosocial workplace initiatives. Further research is needed to identify psychosocial workplace initiatives that may prevent depressive symptoms in employees exposed to workplace sexual harassment by clients or customers. Whether specific workplace initiatives targeting sexual harassment are more effective than general psychosocial organizational changes needs to be examined. Additionally, the results suggested that the association between sexual harassment and depressive symptoms differed for employees harassed by clients or customers compared to those harassed by colleagues, supervisors or subordinates. This result underlines the need to investigate sexual harassment from clients or customers and sexual harassment by colleagues, supervisors or subordinates as two distinct types of harassment.

## Additional file

Additional file 1: Appendix 1. Coding of occupational group. (DOCX 13 kb)

## Abbreviations

CI: Confidence interval
DB07: Dansk Branchekode 2007
DSM-IV: Diagnostic and Statistical Manual of Mental Disorders fifth edition
ICD-10: International Classification of Diseases 10th revision
ISCO-08: International Standard Classification of Occupations approved in 2008
MDI: Major Depression Inventory
N: number
NACE: Statistical classification of economic activities in the European Community
OR: Odds ratio
SAS: Statistical Analysis System
SCAN: Schedules for Clinical Assessment in Neuropsychiatry
WEADW: Work Environment Activities in Danish Workplaces Study
WEHD: Work Environment and Health in Denmark cohort study
