# Supplementary material for: Workplace sexual harassment and depressive symptoms: a cross-sectional multilevel analysis comparing harassment from clients or customers to harassment from other employees amongst 7603 Danish employees from 1041 organizations
Source: BMC Public Health. 2017 Sep 25;17:675. doi: 10.1186/s12889-017-4669-x (PMC5611567; doi:10.1186/s12889-017-4669-x)
Supplement: Additional file 1: — Appendix 1. Coding of occupational group. (DOCX 13 kb) [file 12889_2017_4669_MOESM1_ESM.docx]

**Appendix 1. Coding of occupational group.**

We made the classification to establish large relatively homogeneous groups. Every main group consists of subgroups selected in reference to Dansk Branchekode 2007 (DB07), which is the Danish version of EU’s nomenclature (NACE) (Torma et al., 2007). All enterprises have an activity code based on the main activity. The presented code is a 5-digit code. The first four digits is the NACE code. The first two digits represents the main group, the first three digits the group and the first four digits the sub-group. The last digit indicates the Danish division of the sub-groups.

| Occupational groups | | | | |
| --- | --- | --- | --- | --- |
| **Knowledge work** | **Private service** | **Care work** | **Industrial work** | **Building and construction** |
| 62.00.0 Information technology service activities | 47.00.1 Supermarkets and department stores, etc. | 86.00.1 Hospital activities | 10.00.1 Production of meat and meat products | 41.00.0 Construction of buildings |
| 71.00.0 Architectural and enginee­­ring activities | 49.00.2 Transport by suburban trains, buses and taxi operation etc. | 87.00.0 Residential care activities | 10.00.3 Manufacture of dairy products | 42.00.0 Civil engineering |
| 84.00.1 Public administration | 55.00.0 Hotels and similar accommodation | 88.0.0 Social work activities without accommodation | 10.00.4 Manufacture of grain mill and bakery products | 43.00.9 Bricklaying and other specialized construction activities and site preparation activities |
| 85.00.1 Primary education | 56.00.0 Restaurants |  | 11.00.0 Manufacture of beverages | 43.00.1 Construction installation activities |
| 85.00.2 Secondary education | 64.00.1 Monetary intermediation |  | 21.00.0 Pharmaceuti­cals | 43.00.2 Building completion and finishing |
|  | 81.00.0 Services to buildings, cleaning and landscape activities |  | 22.00.0 Manufacture of rubber and plastic products |  |
|  |  |  | 25.00.0 Manufacture of fabricated metal products |  |
|  |  |  | 28.00.1 Manufactures of engines, windmills and pumps |  |
|  |  |  | 28.00.2 Manufacture of other machinery |  |
|  |  |  | 31.00.0 Manufacture of furniture |  |
